# Environmental heterogeneity and population differences in blue tits personality traits

**DOI:** 10.1093/beheco/arw148

**Published:** 2016-12-20

**Authors:** Gabrielle Dubuc-Messier, Denis Réale, Philippe Perret, Anne Charmantier

**Affiliations:** a Département des Sciences Biologiques, Université du Québec à Montréal, CP-8888 Succursale Centre-ville, Montréal, Québec, Canada and; b Centre d’Écologie Fonctionnelle et Évolutive, Unité Mixte de Recherche CNRS 5175, 1919 Route de Mende, Montpellier Cedex 5, France

**Keywords:** *Cyanistes caeruleus*, life-history strategy, local adaptation, pace-of-life syndrome, personality

## Abstract

Environmental heterogeneity can result in spatial variation in selection pressures that can produce local adaptations. The pace-of-life syndrome hypothesis predicts that habitat-specific selective pressures will favor the coevolution of personality, physiological, and life-history phenotypes. Few studies so far have compared these traits simultaneously across different ecological conditions. In this study, we compared 3 personality traits (handling aggression, exploration speed in a novel environment, and nest defense behavior) and 1 physiological trait (heart rate during manual restraint) across 3 Corsican blue tit (*Cyanistes caeruleus*) populations. These populations are located in contrasting habitats (evergreen vs. deciduous) and are situated in 2 different valleys 25 km apart. Birds from these populations are known to differ in life-history characteristics, with birds from the evergreen habitat displaying a slow pace-of-life, and birds from the deciduous habitat a comparatively faster pace-of-life. We expected personality to differ across populations, in line with the differences in pace-of-life documented for life-history traits. As expected, we found behavioral differences among populations. Despite considerable temporal variation, birds exhibited lower handling aggression in the evergreen populations. Exploration speed and male heart rate also differed across populations, although our results for exploration speed were more consistent with a phenotypic difference between the 2 valleys than between habitats. There were no clear differences in nest defense intensity among populations. Our study emphasizes the role of environmental heterogeneity in shaping population divergence in personality traits at a small spatial scale.

## Lay Summary

The pace-of-life syndrome hypothesis predicts that environmental differences can trigger the evolution of different life-history strategies, including differences in personality. A 5-year study reveals that blue tits breeding in different habitats differ in personality and in life-history characteristics, such as adult survival probability. This is a rare demonstration of personality differences across a small geographical scale, suggesting behavior and life-history traits have coevolved in a local adaptation process.

## INTRODUCTION

Environmental heterogeneity can have a fundamental impact on phenotypic diversity. In particular, heterogeneous environments can result in spatially variable selection pressures, thereby contributing to phenotypic divergence among populations via phenotypic plasticity or via local adaptation (Endler 1986; Kawecki and Ebert 2004; Nosil et al. 2005; Wang and Bradburd 2014). Gene flow among different habitat patches can limit the action of environmental heterogeneity on the evolution of local adaptations, but its impact varies depending on the distance among habitat patches and on the ecology of the species (Lenormand 2002; Kawecki and Ebert 2004; Wang and Bradburd 2014). The evolutionary importance of environmental heterogeneity and gene flow has been highlighted in multiple studies of morphological or life-history traits (Reznick et al. 2001; Garant, Forde, et al. 2007; Garant, Kruuk, et al. 2007; Siepielski et al. 2013). In contrast, their roles in the evolution of behavioral adaptations have seldom been studied (Bell 2005; Quinn et al. 2009; Dingemanse et al. 2010; Dingemanse and Réale 2013), maybe because behavioral traits have often been described as highly plastic. However, we now know that repeatable and heritable behavioral differences among individuals, that is, animal personality, can be found in numerous species (van Oers et al. 2005; Réale et al. 2007; Bell et al. 2009; Dingemanse and Dochtermann 2014). In addition, recent studies in various taxa have shown that personality phenotypes can be under strong selection and their selection regime can fluctuate depending on environmental conditions (Réale and Festa-Bianchet 2003; Dingemanse et al. 2004; Boon et al. 2007; Smith and Blumstein 2008; Quinn et al. 2009; Conrad et al. 2011; Montiglio et al. 2014; Nicolaus et al. 2016). For example, in great tits (*Parus major*), the strength of selection on exploratory behavior varies spatially and temporally according to local density and resource availability (Quinn et al. 2009; Nicolaus et al. 2016). In the common lizard (*Zootoca vivipara*), when population density is low, individuals that are more sociable and less active grow faster and survive longer than less sociable and more active individuals, but these differences disappear at higher density (Le Galliard et al. 2015).

An increasing number of studies are showing that personality traits covary with life-history and physiological traits (Réale et al. 2000; Boon et al. 2007; Dammhahn 2012; Korsten et al. 2013; Montiglio et al. 2014; Careau et al. 2015). Réale et al. (2010) developed the pace-of-life syndrome hypothesis, where they postulated that personality and physiological traits might have (co)evolved with life-history strategies (see also Ricklefs and Wikelski 2002; Wikelski et al. 2003; Wiersma et al. 2007). According to this hypothesis, individuals, populations, or species are positioned along a slow-fast pace-of-life continuum. For example, individuals showing risky behaviors resulting in increased predation probability (e.g., faster exploration patterns, higher aggressiveness, and higher boldness) are positioned on the fast end of the pace-of-life continuum and should therefore reproduce at an earlier age, produce more offspring per reproductive event, and have lower adult survival, whereas those showing safer behaviors (slower exploration, less aggressiveness, and lower boldness) should be at the slow end of the pace-of-life continuum (Réale et al. 2010). Based on the asset-protection principle (Clark 1994), theoretical studies predict that the association between personality traits related to risk-taking and life-history traits might emerge when behavior mediates life-history trade-offs, such as the trade-off between current and future reproduction (Stamps 2007; Wolf et al. 2007). Such an association has been recently observed in a number of empirical studies (Biro and Stamps 2008; Réale et al. 2010; Nicolaus et al. 2012).

The association between personality/life-history traits and fitness may vary in time or in space, depending on the environmental conditions and fitness expectations (Réale et al. 2010; Montiglio et al. 2014). For example, according to the pace-of-life syndrome hypothesis, environmental conditions that reduce residual reproductive value (i.e., low adult survival) should favor a fast life-history strategy (i.e., strong reproductive investment early in life and reduced longevity) and a fast personality (i.e., risky behavior such as fast exploration pattern and high aggressiveness) if the fast personality phenotype favors current reproduction at the expense of future survival. Conversely, environmental conditions that increase residual reproductive value (i.e., high adult survival) but provide limited resources for reproduction should favor the evolution of both a slow pace-of-life (i.e., prolonged longevity and reproductive investment spread over a long lifetime) and a slow personality (i.e., safer behavior such as slow exploration pattern and low aggressiveness). Hence, spatial and temporal variation in environmental conditions has the potential to create a geographical mosaic of a combined set of personality and life-history phenotypes or to promote the evolution of a coordinated phenotypic plasticity for a body of traits (Montiglio et al. 2014). To date, only a few empirical studies have shown that populations inhabiting different habitats differ in suites of traits involved in the pace-of-life syndrome (but see Atwell et al. 2014).

**Figure 1:**
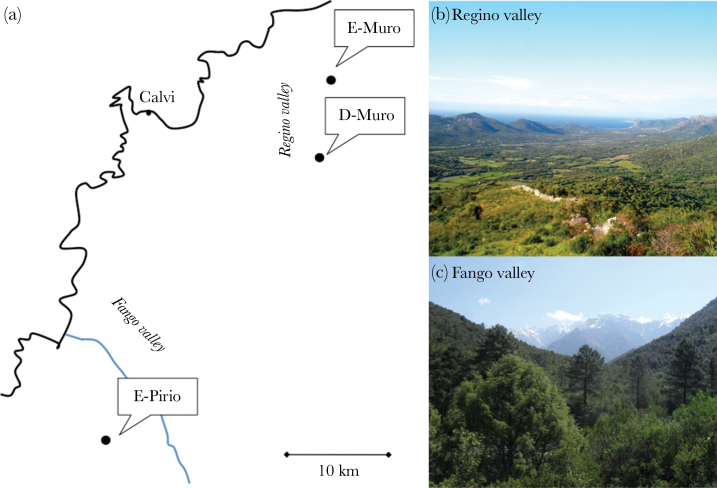
(a) Schematic representation of our 3 study populations located in 2 different valleys: the Fango valley and the Regino valley. The study area in the Regino valley is divided into 2 study populations: E-Muro and D-Muro. E-Muro is located in a forest dominated locally by evergreen oaks and D-Muro in a forest dominated by deciduous oaks. The E-Pirio population is located in a forest dominated by evergreen oaks in the Fango valley; (b) picture of the Regino valley and (c) of the Fango valley.

In this study, we compared the distribution of personality phenotypes across 3 blue tit (*Cyanistes caeruleus*) populations living in contrasting habitats (Figure 1). Long-term monitoring of these populations has previously revealed strong phenotypic differences for numerous life-history, morphological, and ornamental traits despite their spatial proximity (from 5.6 to 25.0 km between each population; Table 1; Charmantier et al. 2016). These populations live in 2 different valleys on the island of Corsica (France) dominated by different tree species, the deciduous downy oak (*Quercus pubescens*) and the evergreen holm oak (*Quercus ilex*). One population is located in a deciduous habitat (Deciduous-Muro, hereafter D-Muro), whereas the other 2 populations are located in a habitat dominated locally by evergreen oaks (Evergreen-Muro and Evergreen-Pirio, hereafter E-Muro and E-Pirio; Figure 1). The deciduous versus evergreen nature of the locally dominant tree species in each population and valley has a cascading influence on several ecological features, which in turn affect the birds’ life-history characteristics (Blondel et al. 1999). For example, large differences in the timing and abundance of food resources (i.e., mainly the leaf-eating *Tortrix viridana* caterpillars) result in differences in clutch size, nestling number and up to a month difference in laying dates between E-Pirio and D-Muro (Table 1; Charmantier et al. 2016). Furthermore, individuals in the 2 evergreen habitats have higher adult survival probabilities than individuals from the deciduous habitat (Table 1). Hence, based on their life-history characteristics, individuals from the evergreen habitats could be characterized as displaying a slower pace-of-life than individuals from the deciduous habitat D-Muro, showing a comparatively faster pace-of-life (Table 1).

**Table 1:** Life-history, morphological characteristics (mean [*n*]), and caterpillar abundance in the 3 Corsican blue tit populations studied (France)

Habitat/populations	Deciduous D-Muro	Evergreen E-Muro	Evergreen E-Pirio
First year of monitoring	1993	1998	1976
Annual adult survival probability^a^	0.39 (15)	0.58 (15)	0.47 (15)
Date of first egg laying^b^, 1 = 1 March	38.56 (1233)	48.21 (640)	70.08 (1920)
Male body mass (g)^b^	9.82 (1032)	9.66 (1032)	9.37 (1607)
Female body mass (g)^b^	9.66 (1153)	9.47 (480)	9.23 (1616)
Male tarsus length (mm)^b^	16.52 (578)	16.42 (198)	16.27 (789)
Female tarsus length (mm)^b^	16.05 (614)	15.99 (224)	15.84 (798)
Clutch size^b^	8.50 (1235)	7.12 (638)	6.61 (1913)
Number of fledglings^b^	6.60 (1092)	4.14 (557)	4.15 (1273)
Caterpillar abundance^c^	762.87	689.32	87.10
Pace-of-life	Fast	Slow/intermediate	Slow

At the bottom of the table, we have indicated the pace-of-life syndrome characterizing each population according to their life-history characteristics.

a Dubuc-Messier G et al. (in preparation): these survival probabilities were estimated from fully time-dependent models with the software E-SURGE v.1.9 (Choquet et al. 2009; from years 2000 to 2015); they are in line with the survival probability estimations of Grosbois et al. (2006; Pirio: years 1985–2000 and D-Muro: 1993–2000); the number in parenthesis refers to the number of years considered in the analyses.

b Charmantier et al. (2016) (collected between the first year of monitoring and 2014).

c Mean maximal frass mg/m^2^ per day (sampled in each population between 2011 and 2015 during the breeding period using 0.25 m^2^ trays placed under the forest canopy and collected twice a week, see Zandt et al. (1990) for details about the sampling procedure).

In each population, we repeatedly measured 3 behavioral traits and 1 physiological trait traditionally used in personality studies and that are related to risk taking. First, we measured docility as the reaction of birds toward the handler (Réale et al. 2007). Docility is related to life-history traits (Réale et al. 2000, 2009) and has been shown to be repeatable and heritable in blue tits (Brommer and Kluen 2012; Class et al. 2014). Although our test was slightly different than Brommer and Kluen (2012), we decided to call docility “handling aggression”, so that the highest score for this behavior corresponds to the most aggressive response and to facilitate comparisons with other works on blue tits (Brommer and Kluen 2012; Class et al. 2014). Second, we quantified heart rate during manual restraint. This trait has been shown to be repeatable in different species (Koolhaas et al. 1999; Réale et al. 2009; Montiglio et al. 2012; Ferrari et al. 2013) and is also potentially associated with life-history characteristics (Réale et al. 2010). Heart rate during stressful events, such as manipulation, is often studied in the context of coping styles and is linked to the activity of the sympathetic and parasympathetic nervous systems (Koolhaas et al. 1999; Ferrari et al. 2013). Coping styles correspond to the way individuals cope with a stressful situation (Koolhaas et al. 1999; Groothuis and Carere 2005). Coping style is linked with many of the behaviors typically associated with fast and slow pace-of-life (Réale et al. 2010): at one extreme, proactive individuals are highly active, highly aggressive, and fast explorers, whereas at the other extreme, reactive individuals are, lowly active, lowly aggressive, and slow explorers (Koolhaas et al. 1999). The sympathetic nervous system is presumed to be the dominant system in proactive individuals, whereas the parasympathetic nervous system is presumed to be dominant in reactive individuals (Koolhaas et al. 1999, 2007). Third, we quantified exploration behavior in a novel environment. Exploration in a novel environment is traditionally used in personality studies (Réale et al. 2007) and is repeatable in blue tits (Mutzel et al. 2013). Finally, we measured nest defense behavior, which is assumed to decrease the probability that a predator will harm the offspring in a nest while increasing the probability of injury for the parents (Trivers 1972; Montgomerie and Weatherhead 1988). Nest defense behavior involves a trade-off between parental survival and offspring protection. An increasing number of studies are showing among-individual variation in nest defense intensity revealing among-individual differences in investment in current reproduction versus residual reproductive value (Montgomerie and Weatherhead 1988; Hakkarainen and Korpimäki 1994; Kontiainen et al. 2009; Fresneau et al. 2014).

We expected that the difference in ecological conditions between habitats and populations would produce different selection pressures on personality traits that would result in different mean personality phenotypes among habitats and populations in our study system. As proposed by the pace-of-life syndrome hypothesis, we expected that the differences in personality phenotype between habitats would be linked to their difference in life-history characteristics. More specifically, we predicted that in the evergreen habitats individual blue tits would display personality phenotypes associated with a slow pace-of-life, whereas individuals inhabiting the deciduous habitat would display personality phenotypes associated with a faster pace-of-life. Based on previous studies on personality, we expected that a higher handling aggression (Réale et al. 2010), a faster heart rate during manual restraint (increased activity of the sympathetic system; Koolhaas et al. 1999; Ferrari et al. 2013), a faster exploration pattern (Réale et al. 2010; Nicolaus et al. 2012), and a higher nest defense intensity (Montgomerie and Weatherhead 1988) would be associated with a faster life style and would therefore be found in individuals located in the deciduous population (D-Muro). Because the differences in ecological conditions and life-history characteristics are more salient between E-Pirio and D-Muro than between E-Muro and D-Muro (Table 1), we expected more substantial differences in personality phenotypes between the 2 former populations. In addition, because sex differences in personality traits and behavioral syndromes are found in an increasing number of studies (Schuett et al. 2010; Dammhahn 2012; Fresneau et al. 2014), we expected to find sex differences in mean phenotypes and sex-specific differences between populations.

The 2 valleys (Regino and Fango) have different ecological conditions, which could also be important in shaping the personality phenotype of blue tits in each population. Indeed, in the Regino valley (E-Muro and D-Muro populations; Figure 1), the dominant tree species is the deciduous oak, and the evergreen oak is only present in isolated patches (e.g., E-Muro). In contrast, in the Fango valley (E-Pirio), the deciduous oak is completely absent, and the evergreen oak is the dominant species and forms a homogeneous habitat (Porlier et al. 2012). The 2 valleys also differ in their level of anthropogenic activities, precipitation, and temperature. Therefore, the 2 evergreen populations share the same dominant oak species at a small spatial scale but differ in their ecological conditions at the scale of the valley. Although E-Muro and D-Muro share the same large-scale ecological conditions that are specific to the valley, they experience different ecological conditions at a small spatial scale (e.g., proportion of deciduous oak). The results from previous studies in this system suggest that morphological and life-history traits are shaped by factors that vary at different spatial scales; some traits are shaped by factors specific to the local dominant oak species and others by factors specific to the valley. For example, E-Muro birds display laying dates and female tarsus lengths similar to D-Muro but their average number of fledglings and adult survival probabilities are more similar to E-Pirio (Table 1). Hence, the comparison of personality phenotypes among these 3 populations may provide insight into the spatial scale at which environmental conditions affect the phenotype of different personality and physiological traits.

## METHODS

### Study species, sites, population characteristics, and field protocol

Blue tits are small (9–13g), forest cavity-nesting passerine birds, commonly found in wooded habitats of the western Palearctic, ranging from southern Scandinavia to the Canary Islands. Blue tits are socially monogamous with biparental care and are sedentary in our study populations. This study was conducted in 3 populations in the region of Calvi, Corsica, France: E-Pirio (42°34′N, 08°44′E; 200-m elevation; 205 nest-boxes distributed in 2 study plots), E-Muro (42°35′N, 08°57′E; 100 m elevation; 75 nest-boxes distributed in 3 study plots), and D-Muro (42°32′N, 08°55′E; 350 m elevation; 110 nest-boxes distributed in 3 study plots). These populations have been studied since 1976, 1998, and 1994, respectively (Blondel et al. 2006; Charmantier et al. 2016).

A weekly to daily monitoring over the course of the breeding season, from early April to the end of June, allowed us to record the exact laying dates and clutch sizes for all broods in nest-boxes. Adult blue tits were captured in nest-boxes, identified or ringed with unique metal rings provided by the Centre de Recherches sur la Biologie des Populations d’Oiseaux (CRBPO, Paris, France), and weighed to the nearest gram using a Pesola® spring. In 2014 and 2015, we used color rings (Ecotone® 2.7mm) placed on the tarsus for further identification during nest defense observations. We determined the sex of each individual by examining the presence/absence of a brood patch during the breeding period or based on feather coloration outside the breeding period (Perrins 1979; Ferns and Hinsley 2010; Fresneau et al. 2014). All nestlings were also weighed, measured, and uniquely identified with metal rings placed on their right tarsus at 9–15 days of age.

### Behavioral tests

Behavioral tests were run between 2011 and 2015. Tests were performed either during the prebreeding period when males and females paired up and started defending a territory (from 17 to 30 March for D-Muro and E-Muro and from 4 April to 3 May for E-Pirio) or during the breeding period when adults were feeding nestlings. During the prebreeding period, birds were caught with a mist net or lured into a trap using a live blue tit decoy and playback of territorial calls near a nest-box. Birds were then tested for handling aggression, heart rate during manual restraint, and exploration behavior in a novel environment. During the breeding period, we also measured handling aggression on parents caught inside the nest-box when nestlings were 10–14 days old and measured nest defense behavior when nestlings were 9 days old. All tests, except handling aggression, were performed only once a year for each individual. Occasionally, a bird was caught twice during the prebreeding period, but it was immediately released after the second capture, or if a test was done unintentionally, this test was discarded from the data set (tests from 22 individuals were discarded). Handling aggression tests were done at most twice per year per individual (once during the prebreeding and once during the breeding period). For every trait, there were 1 or 2 observers who performed most of the observations in every population for at least 3 years (40–70% observations were made by 2 observers, see Supplementary Tables S1–S3 for details).

### Handling aggression score

Handling aggression was scored from 2011 to 2014. The test was done within 2 min after capture, directly after removing the bird from the trap and prior to any other manipulation. The handler held the bird in the upright position, head up, with his back facing the handler. He held the bird with one hand and placed the bird’s legs between his forefinger, his middle finger, and his thumb to let the bird free to move its tail and wings. The handler pointed the forefinger of his other hand at a spot about 2–3cm in front of the bird’s beak and noted if the bird struck at his finger, and the position of its wings and tail. After 2 s in this position, the handler moved his forefinger toward the bird’s beak 2 or 3 times and recorded its reaction. The score ranged from 0 (the bird shows no reaction) to 3 (the bird spontaneously strikes the handler’s fingers and spreads its wings and tail). The scoring protocol is reported in detail in Supplementary Table S4. The entire test lasted less than a minute.

### Heart rate during manual restraint

Heart rates during manual restraint (HR, hereafter) were collected between 2011 and 2015. Following the handling aggression scoring, the bird was put in a cloth bag and brought to the novel-environment apparatus (approximately 1–200 m away) where we recorded heart rate during manual restraint. Prior to recording, the handler placed the bird’s head between his forefinger and his middle finger and put the bird’s legs between his thumb and forefinger. HR was then recorded for 30s, using a digital recorder with the microphone placed close to the bird’s cloaca and directed toward the heart.

Back in the lab, we used the software Avisoft SASLab Pro version 5.1 to extract the mean time interval (seconds) between 2 heart beats using approximately 100 consecutive heart beats per individual. We used the number of heartbeats in a minute (60/mean time interval) in the analysis. We recorded HR instead of breath rate (BR hereafter), a measure more commonly used in bird studies (Carere and van Oers 2004; Fucikova et al. 2009; Brommer and Kluen 2012; Kluen et al. 2014), because although the analysis is more time consuming, HR scoring can be automated and is thus less prone to errors or biases than BR. To compare our results with other studies on birds, we examined the correlation between HR and BR on a subsample of 102 birds in 2015. BR was measured right after recording HR, following the protocol described by Brommer and Kluen (2012). In short, we measured the time required for the bird to take 30 breaths and repeated this procedure twice. We transformed the average of the 2 measures to obtain the number of breaths in a minute (1800/average of the 2 measures).

### Exploratory behavior in a novel environment

Data on exploration were collected between 2011 and 2014. After a bird’s heart rate was measured, it was placed in a novel-environment apparatus built on the model proposed by Mutzel et al. (2013). From 2011 to 2013, the apparatus consisted of a large white cage (120cm × 50cm × 50cm) with 6 perches and one side composed of small mesh, allowing us to video trials (Supplementary Figure S1a). The apparatus was placed in the trunk of a car (Kangoo, Renault), and the car side and back windows were covered with a tarp to isolate the tested bird from the external environment (Supplementary Figure S1a). Natural light was used for the video recording. In 2014, to homogenize light conditions over time and space, we used a slightly smaller novel-environment apparatus (110×50×50cm) placed inside a closed trailer and artificial lights for every trial (Supplementary Figure S1b). Prior to all trials, the bird was placed for 2min in a closed chamber (15×15×15cm) located on the right-hand side of the novel-environment apparatus and connected to the main chamber by a sliding door. We then opened the door, gently pushed the bird inside the main chamber and video recorded its behavior for 5min. The bird was subsequently retrieved of the novel-environment apparatus, ringed when necessary, weighed, and released. Birds that could not be put in the novel-environment apparatus right after the heart rate measurement were placed in a small cloth bag for a maximum of 30min. When the time interval between the HR recording and the novel-environment trials was more than 30min, the birds were placed in a cage with water and mealworms (*n* = 193 trials).

Back in the lab, we extracted the average speed of the bird (centimeter/second) during the trial using the software EthoVision XT version 9, and we used this variable in the analyses as a measure of exploratory behavior. Compared with other ways of measuring movements in the novel environment, the computation of average exploration speed can be automated, reducing both errors and biases. Furthermore, the average speed was well correlated to the number of large flights in our novel-environment apparatus (*r* = 0.9, *P* < 0.001, *n* = 20), a measure that has commonly been used to quantify exploratory behavior in other studies (Dingemanse et al. 2002; Mutzel et al. 2013).

### Nest defense behavior

Nest defense trials were conducted from 2012 to 2015 in E-Pirio and in 2012, 2014, and 2015 in D-Muro and E-Muro. We measured nest defense behavior with a stuffed Eurasian jay (*Garrulus glandarius*), a common predator of blue tit nestlings in Corsica. The decoy was placed as close as possible to the nest-box (minimum = 0.50 m, maximum = 4.00 m, mean = 1.18 m, standard deviation [SD] = 0.67 m). The observer was hidden between 6 and 30 m from the nest-box (mean = 14.14 m, SD = 4.33 m). As soon as a parent blue tit approached within 15 m of the nest-box, we estimated its minimal approach distance from the nest-box during the next 5min. For practical reasons, recording the distance to the nest-box was much easier and more accurate than estimating distance to the predator, but the 2 distances were highly correlated and parents did not approach nest-boxes with the intention of feeding their nestlings because they systematically dropped or ate their prey once they located the predator, and none entered the nest-box during the test (Dubuc-Messier G, personal observation). We recorded the behavior of the 2 partners at the same time when they were both present. Tests were done only once per nest-box per year. Birds that did not enter the 15 m perimeter within 15min after the beginning of the test (decoy in place) were discarded from the data set. The sex and identity of each parent was determined using individual color rings, the position of the metal ring (adult females are ringed on their left leg), or based on feather coloration. Birds were caught for final identification and/or ringing the day after the test.

### Statistical analysis

To test for phenotypic differences among populations, we used univariate linear mixed-models and included population in fixed effect. Models were run separately for each trait. In all models, we also included sex, age (juvenile or adult), year, and the 2-way interactions between year and population and between sex and population as fixed effects. The time of the day when each test was performed (hour) was also added as a confounding variable for each trait. The random effect structure of each model is detailed at the end of this section.

Relevant confounding and biological variables were added for each trait. For handling aggression score, because this trait was repeatable within a year and for a given period of captures across years (Supplementary Table S5), we pooled the data from both periods and added “capture period” as a fixed effect.

For HR, because there was substantial among-individual variation in the time between capture and HR recording (minimum time: 0min; maximum time: 96min), we added the time between capture and recording as a fixed effect in the analyses. We also included body mass as a fixed effect for this trait because HR is related to metabolic rate and both traits are positively correlated with body mass (Green 2011). We investigated the relationship between HR and BR using a univariate linear model. We used HR as a response variable, mean BR as a fixed effect and included in fixed effect all the significant confounding variables for HR identified previously.

For average exploration speed, we also included as confounding variables the time interval between capture and trials (minimum = 5min, maximum = 57min, mean = 21.15min, SD = 11.40min) and the confinement system used between heart rate recording and trial (3 classes: no confinement, bag, or cage).

For nest defense behavior, we added as confounding variables the distance between the decoy and the nest-box, the distance between the observer and the nest-box, and the identity of the decoy (we used 2 different stuffed Eurasian jays). To ensure that any difference between populations would not be caused by the availability of perches close to the nest-box, we included the distance from the nest-boxes to the closest branch as a confounding variable in all models. However, this variable was recorded for all years in E-Pirio (2012–2015) but only for 2014 and 2015 in D-Muro and E-Muro. The inclusion of this variable in the models thus limited our population comparison to 2014 and 2015. We also tested for a correlation between the nest defense behavior of an individual and its partner’s behavior during the test using a univariate linear model. We used female minimal approach distance as a response variable and male minimal approach distance as a fixed effect and included as fixed effect all the significant confounding variables for nest defense. Minimal approach distance was square root transformed prior to analyses.

To control for differences in reproductive status among individuals during trials, we used the time between measurement and laying date as an additional fixed effect for HR and exploration speed. For handling aggression, the “capture period” fixed effect and the time between measurement and laying date were highly correlated, we thus kept only “capture period” in models. We did not control for the reproductive status of individuals for nest defense behavior because all trials were performed when nestlings had 9 days old.

To control for any effect of habituation of the birds in response to either repeated manipulations by humans or to repeated visits in the novel-environment apparatus, we used the order of capture (for handling aggression and HR) or the order of the novel-environment trials as a fixed effect in the models. We assumed that there was no habituation during nest defense trials, because this test imitated a real predator attack and trials were done only once a year for a given individual.

The significance of the confounding variables was first tested using likelihood-ratio tests (*L*-ratio test; Bates et al. 2014b) and a backward stepwise procedure starting with a model including all the confounding variables. We then used the same procedure to test for the significance of the biological variables (population, age, sex, and year) starting with a model containing all the biological variables and the significant confounding variables. All models were run first on a data set combining both sexes. When a significant interaction between sex and population was found, we ran separated models for males and females using the fixed effect structure of the models selected with the sexes pooled.

If a significant population effect was revealed for a trait, we tested for a significant difference between 2 given populations by including 2 populations in a single model (E-Pirio and D-Muro; E-Pirio and E-Muro; E-Muro and D-Muro) and by running *L*-ratio tests to test for the presence of a significant population effect. In these models, we did not include the interaction between year and population or between sex and population. This allowed us to test the significance of the population term alone. Comparing a model with the interaction term between population and year or population and sex to a model without the term population would test simultaneously for 2 effects: the interaction between the 2 terms and the population. In addition, not including the interaction between population and year or sex allowed us to investigate the difference in phenotype between populations over the entire study period not only for one specific year or sex. We also checked for a significant valley effect (Regino vs. Fango) rather than a population effect and present these results in Supplementary Table S12.

We included individual and observer identity as random effects to decompose the phenotypic variance into among-individual (*V*_ID_), among-observer (*V*_OBS,_ not included for average exploration speed in the novel environment), and residual (*V*_R_) components and to account for the nonindependence of repeated measures on the same individual. Repeatability of personality traits was estimated using repeated behavioral trials for the same individuals across years. We calculated adjusted repeatability as *r*_ID_ = *V*_ID_/(*V*_ID_ + *V*_R_) or *V*_ID_/(*V*_ID_ + *V*_OBS_ + *V*_R_) using the fixed effect structure selected previously and agreement repeatability as *r*_ID_ = *V*_ID_/(*V*_ID_ + *V*_R_) using no fixed effects (Nakagawa and Schielzeth 2010). We calculated the repeatability of each trait for the metapopulation and for each population and sex, separately. We calculated the observer effect as *V*_OBS_/(*V*_ID_ + *V*_OBS_ + *V*_R_) using the fixed effect structure selected previously. We assessed the significance of the random terms using *L*-ratio tests (Pinheiro and Bates 2000).

All analyses were done using the package *lme4* (Bates et al. 2014a) in R (version 3.1.3, R Core Team 2015). Confidence intervals (CI) were generated using the *confint.merMod* function of the *lme4* package.

Captures were performed under ringing permits delivered by the CRBPO (ringing permit number 1907 to A.C., program permit number 369). All experimental protocols described here were approved by the ethics committee for animal experimentation of Languedoc Roussillon (305-CEEA-LR-12066 approved in 2012), by Regional Institutions (bylaw issued by the Prefecture on 15/06/2012 n° 2012167-0003), and by the Comité Institutionnel de Protection des Animaux (UQAM; CIPA-769-2015; 0413-R1-769-0414).

## RESULTS

### Repeatability

Significant among-individual differences were observed for each trait, with adjusted repeatability estimates ranging from 0.26 to 0.75 (Table 2). Observer identity significantly affected handling aggression (proportion of total variance = 0.03; *L* ratio = 21.33; *P* < 0.001; 17 observers) and the minimum approach distance (0.27; *L* ratio = 31.60; *P* < 0.001; 7 observers), but not HR (*L* ratio = 0; *P* = 0.99; 5 observers). Handling aggression, HR, and average exploration speed were significantly repeatable for all populations except for exploration speed in E-Pirio, where a large proportion of the variance was nevertheless explained by bird identity (Supplementary Table S6). The small number of repeated measures for nest defense behavior prevented us from testing its repeatability in each population separately. All traits were repeatable for both sexes except nest defense behavior, which was repeatable for females only (Supplementary Table S7).

**Table 2:** Among-individual, among-observer, and residual variances (95% CI) along with adjusted and agreement repeatability (*r*_ID_; Nakagawa and Schielzeth 2010), sample sizes, and statistics for the significance of adjusted repeatability for 3 personality traits and 1 physiological trait measured in 3 Corsican blue tits populations (France)

Trait	*V*_ID_ (CI)	*V*_OBS_ (CI)	*V*_R_ (CI)	*r*_ID_ adjusted; agreement (*N*_ind_ 1, 2, 3, 4, +)	*L* ratio	*P* value
Handling aggression	0.22 (0.16; 0.28)	0.03 (0.01; 0.07)	0.61 (0.55; 0.67)	0.26; 0.30 (458, 242, 114, 66, 33)	82.39	<0.001
HR (beats/min)	7103 (5396.17; 8972.43)	201.20 (0.00; 458.88)	2326 (1655.02; 3411.38)	0.75; 0.64 (243, 34, 18, 4, 1)	41.25	<0.001
Average exploration speed (cm/s)	22.74 (17.11; 33.83)	n/a	25.34 (12.20; 32.10)	0.47; 0.40 (385, 89, 19, 1, 0)	17.10	<0.001
Nest defense (m)	0.15 (0.06; 0.23)	0.14 (0.03; 0.44)	0.22 (0.14; 0.30)	0.30; 0.52 (196, 31, 7, 0, 0)	9.37	<0.005

(*N*_ind_ 1, 2,3,4, +) indicates how many individuals were included in the models with 1, 2, 3, 4 or more than 4 tests. *L* ratio and *P* values are from the comparison of a full model and a model without the term individual identity as random effect. Data from the 3 populations and for both sexes are included. *V*_ID_, *V*_OBS_, *V*_R_, and adjusted repeatability were calculated from models with all the significant fixed effects for each trait; for details on fixed effects structure and effect sizes, see Supplementary Tables S8–S11. n/a, not applicable.

### Population difference and variation across sex and time

#### Handling aggression

**Figure 2:**
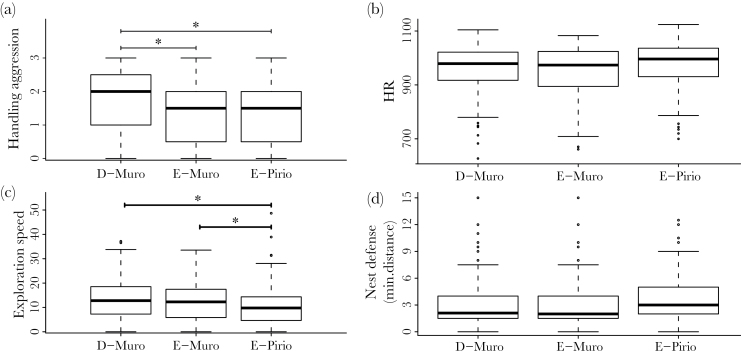
Boxplots for (a) handling aggression (D-Muro: number of observations [*n*] = 703; E-Muro: *n* = 447; E-Pirio: *n* = 549); (b) heart rate during manual restraint (HR in beats/min; D-Muro: *n* = 143; E-Muro: *n* = 116; E-Pirio: *n* = 107); (c) average exploration speed (speed in cm/s; D-Muro: *n* = 175; E-Muro: *n* = 100; E-Pirio: *n* = 105); and (d) minimal approach distance during nest defense (distance in m; D-Muro: *n* = 75; E-Muro: *n* = 63; E-Pirio: *n* = 147), in 3 blue tit populations in Corsica (France). Male and female data are pooled. The significance of the between-population differences was assessed with models contrasting 2 populations at a time with fixed effects structures as detailed in Table 3 and Supplementary Tables S8–S11 but without the interaction term between year and population; “*” indicates a significant difference (*P* < 0.05) between 2 populations.

Populations differed significantly in average handling aggression score (Tables 3 and 4, Figure 2a, and Supplementary Table S8). Birds in D-Muro (mean = 1.69; SD = 0.95) had a significantly higher handling aggression score than those from E-Muro (mean = 1.48; SD = 0.96) and than those from E-Pirio (mean = 1.49; SD = 0.99), whereas birds in E-Pirio and E-Muro displayed similar scores (Table 4, Figure 2a). Females were less aggressive than males (estimate: −0.34 [95% CI: −0.44; −0.24]; Supplementary Table S8). There was no significant interaction between sex and population for this trait (*P* = 0.31; *L* ratio: 2.35), but there was a significant interaction between population and year; individuals from D-Muro were more aggressive compared to individuals in E-Pirio in 2011, whereas in 2012 and 2013, individuals in D-Muro were less aggressive (Table 3; Supplementary Figure S2a and Table S8).

**Table 3:** Population differences and significant biological variables for 3 personality traits and 1 physiological trait across 3 Corsican blue tit populations (France)

Trait	Fixed effect	*L* ratio	*P* value
Handling aggression	Population	13.84	<0.001
	Year	22.88	<0.001
	Population × year	67.18	<0.001
	Sex	42.33	<0.001
HR (beats/min)	Population	4.15	0.15
	Year	7020.3	<0.001
	Sex	92.60	<0.001
	Body mass	398.94	<0.001
	Population × year	19.16	<0.050
Average exploration speed (cm/s)	Population	13.37	<0.010
	Year	81.91	<0.001
	Sex	14.74	<0.050
	Population × year	8.35	<0.001
Nest defense (m)	Year	16.34	<0.001
	Sex	14.35	<0.001

*L* ratio and *P* values are from the comparison of a full model and a model without the variable of interest. Confounding variables included in the models are described in Supplementary Tables S8–S11. Random effect structures are individual and observer identity. All effect sizes and CI are described in Supplementary Tables S8 and S11.

**Table 4:** Differences in handling aggression score and average exploration speed (cm/s) between pairs of blue tit populations in Corsica (France)

Trait	Populations compared	Estimate (95% CI)	*L* ratio	*P* value
Handling aggression	**E-Pirio versus D-Muro**	−0.14 (−0.27; −0.01)	4.268	**<0.05**
	E-Pirio versus E-Muro	0.11 (−0.04; 0.25)	2.110	0.25
	**E-Muro versus D-Muro**	**−0.28 (−0.41; −0.15**)	**17.752**	**<0.001**
Average exploration speed	**E-Pirio versus D-Muro**	**−3.41 (−5.33; −1.49**)	**10.444**	**<0.005**
	**E-Pirio versus E-Muro**	**−2.01 (−3.97; −0.05**)	**4.324**	**<0.05**
	E-Muro versus D-Muro	−1.27 (−3.30; 0.78)	4.600	0.100

Significant differences between populations are in bold. The second population in the second column is the reference population. Estimates and 95% CI are from a model with the 2 populations of interest and the same fixed effects structure detailed in Table 3 and Supplementary Tables S8 and S10 but without the interaction term between population and year. *L* ratio and *P* values are from the comparison of a full model and a model without the term population as fixed effect.

#### Heart rate

Mean HR during manual restraint was positively related to BR (estimate: 0.82 [95% CI: 0.06; 1.66]; *L* ratio = 4.33; *P* < 0.05): individuals with a fast heart rate breathed faster during restraint. When we did not control for body mass, birds from E-Pirio had a faster HR (mean = 976.24 beats/min; SD = 86.99) than birds from D-Muro (mean: 963.30 beats/min; SD = 87.80) and E-Muro (mean = 955.97 beats/min; SD = 89.18), but birds from E-Muro and D-Muro had a similar heart rate (E-Pirio vs. D-Muro: estimate: 26.64 [95% CI: 1.15; 52.06]; *L* ratio = 3.90; *P* < 0.05; E-Pirio vs. E-Muro: estimate: 30.66 [95% CI: −0.418; 61.71]; *L* ratio = 3.74; *P* = 0.053; D-Muro vs. E-Muro: estimate: −9.09 [95% CI: −36.14; 17.92]; *L* ratio = 0.44; *P* = 0.53). There was also a significant interaction between population and year (*L* ratio = 21.92; *P* < 0.01; Supplementary Figure S2b).

Lighter individuals had a faster HR (*P* < 0.001; Supplementary Table S9), and there was a significant difference in body mass between populations: birds from E-Pirio were lighter than birds from D-Muro (estimate: −0.15 [95% CI: −0.27; −0.05]; *L* ratio = 7.42; *P* < 0.01) and from E-Muro (estimate: −0.11 [95% CI: −0.23; 0.01]; *L* ratio = 3.11; *P* = 0.078). Consequently, mean HR did not differ significantly among populations when we controlled for body mass (E-Pirio vs. D-Muro: *L* ratio = 2.07; *P* = 0.15; E-Pirio vs. E-Muro: *L* ratio = 2.01; *P* = 0.16; E-Muro vs. D-Muro: *L* ratio = 0.308; *P* = 0.58; Figure 2b). There was also a significant interaction between population and year when we controlled for body mass (Table 3; Supplementary Figure S2b and Table S9).

**Figure 3:**
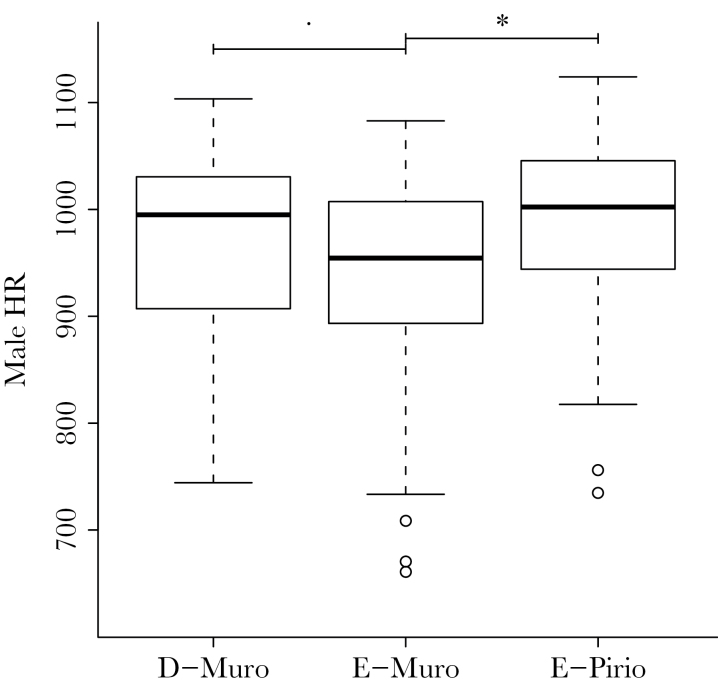
Boxplots representing male heart rate during manual restraint (HR; in beats/min) in 3 Corsican blue tit populations (France; D-Muro: *n* = 62; E-Muro: *n* = 57; E-Pirio: *n* = 48); the significance of the between population difference was assessed with models contrasting 2 populations at a, time with fixed effects structures as detailed in Supplementary Table S9 but without the interaction term between year and population. “.” indicates a marginally significant difference (0.10 > *P* > 0.05), and “*” indicates a significant difference (*P* < 0.05) between 2 populations.

We found a marginally significant interaction between sex and population (*L* ratio = 5.65; *P* = 0.059). When we analyzed both sexes separately and controlled for body mass, males from E-Pirio had a faster HR than males from E-Muro (estimate: 81.03 [95% CI: 35.66; 129.95]; *L* ratio = 11.62; *P* < 0.001) and males from E-Muro had a marginally significantly slower HR than males from D-Muro (estimate: −32.71 [95% CI: −69.68; 3.90]; *L* ratio = 3.08; *P* = 0.079; Figure 3). However, there was no difference in male HR between D-Muro and E-Pirio (*L* ratio = 2.36; *P* = 0.12) and no population effect for females (*L* ratio = 0.90; *P* = 0.65).

#### Average exploration speed

We found a significant population effect for average exploration speed (Table 3): individuals from E-Pirio (mean = 10.37cm/s; SD = 7.49) were significantly slower in their exploration than individuals from D-Muro (mean = 13.52cm/s; SD = 8.39) and E-Muro (mean = 11.84cm/s; SD = 7.16), whereas birds from D-Muro and E-Muro did not differ (Table 4; Supplementary Table S10 and Figure 2c). Females were slower in the novel environment than males (estimate: −2.02 [95% CI: −3.49; −0.55]; Supplementary Table S10), but there was no significant interaction between sex and population (*L* ratio = 0.73; *P* = 0.69). We also found an interaction between population and year for this trait (Table 3). This significant interaction was mainly attributable to 2014 when the difference between D-Muro and E-Pirio was smaller than for the other years (Supplementary Figure S2c).

#### Nest defense behavior

There were no significant difference in nest defense between populations (*L* ratio = 1.85; *P* = 0.40; Figure 2d) and no interaction between population and year for this trait (*L* ratio = 1.92; *P* = 0.38). We found a significant effect of the distance between the closest branch and the nest-box on nest defense intensity. The inclusion of the distance from the nest-box to the closest branch as a fixed effect limited our population comparisons to 2014 and 2015. However, this limitation did not hinder our capacity to detect population differences because there was no significant difference between populations even when we did not include the distance to the closest branch in the model and hence included year 2012 in the comparison (*L* ratio = 2.89; *P* = 0.24). We also found a significant sex-difference for this trait: females had longer minimal approach distances than males (estimate: 0.20 [95% CI: 0.05; 0.35]). Partners’ nest defense behaviors were significantly correlated (estimate = 0.06 [95% CI: −0.06; 0.19]; *L* ratio = 122.48; *P* < 0.001).

## DISCUSSION

This study reveals that blue tits from contrasting habitats display different mean personality phenotypes. In addition, some of our results are consistent with the pace-of-life syndrome hypothesis because birds from the deciduous population D-Muro had a faster phenotype on average (faster exploration pattern and higher handling aggression scores) than birds from the evergreen populations E-Muro and E-Pirio (Table 4, Figure 2). A small number of studies have compared the personality phenotypes of wild populations that differ in ecological contexts (Fraser and Gilliam 1987; Bell and Stamps 2004; Bell 2005; Quinn et al. 2009; Dingemanse et al. 2010; Korsten et al. 2010; Dingemanse et al. 2012). To our knowledge, most of these studies have compared the personality phenotype of a single trait (Fraser and Gilliam 1987; Korsten et al. 2010), the behavioral syndrome structure (Bell and Stamps 2004; Bell 2005; Dingemanse et al. 2010), or the plasticity (Dingemanse et al. 2012) of populations. Very few studies have compared explicitly the personality phenotypes of populations that exhibit different life-history characteristics and that differ in ecological conditions and residual reproductive value in the framework of the pace-of-life syndrome hypothesis. The long-term monitoring of these blue tit populations that display pronounced phenotypic variation on many morphological, life-history, and behavioral traits at a small spatial scale (Charmantier et al. 2016) was an ideal opportunity to test for personality differences in the context of the pace-of-life syndrome.

According to the pace-of-life syndrome hypothesis, populations experiencing different ecological conditions, in particular differing in adult mortality rates, should show different personality phenotypes. More precisely, in the presence of a trade-off between current and future reproduction, theoretical models predict that individuals that have lower residual reproductive value (or asset) should display riskier behavior if it favors current reproduction over future reproduction (Wolf et al. 2007; Sih et al. 2015). In the deciduous population of D-Muro, adult survival is lower than in E-Pirio and E-Muro (Table 1; Grosbois et al. 2006). Because of this lower adult survival, birds inhabiting the deciduous habitat have a lower residual reproductive value. We were thus expecting that birds from D-Muro would show a personality phenotype associated with risk taking and typical of a faster pace-of-life (higher handling aggression score, faster heart rate, faster exploration pattern, and higher nest defense intensity; Clark 1994; Groothuis and Carere 2005; Réale et al. 2010; Cole and Quinn 2014; Sih et al. 2015). As predicted, birds from D-Muro had a faster exploration pattern across all years than birds from the evergreen population E-Pirio (Table 4, Figure 2c). Our results are also consistent with our predictions for handling aggression scores, as birds from D-Muro had a higher handling aggression score overall than birds from E-Muro and from E-Pirio (Table 4, Figure 2a).

Nevertheless, some of our results are not consistent with our predictions. Indeed, there was no population difference in nest defense intensity, and males from E-Pirio had a faster HR than males from E-Muro. In addition, our analyses revealed very strong temporal variation in the differences between populations in handling aggression scores, with patterns that are reversed between years. These results suggest that other factors than the local dominant oak species and the residual reproductive value might be important in shaping the personality phenotype of these blue tit populations.

### A matter of scale

Our study design provides insight into the factors and the spatial scales that shape the phenotypes of different personality and physiological traits. For example, exploration behavior differs between birds from the Regino and Fango valleys (Supplementary Table S12) but did not differ between birds with different local ecological conditions in the same valley (D-Muro and E-Muro birds; Figure 2c and Table 4). These results suggest that processes occurring at the landscape scale (i.e., the valley; proportion of deciduous oak in the surroundings, level of anthropogenic activities, precipitation, and temperature) might be more important in shaping exploration patterns than processes resulting from local ecological conditions occurring at a smaller spatial scale. In contrast, we did not find a significant difference in handling aggression score between the 2 valleys (Supplementary Table S12), but we did find differences between populations with different small-scale ecological conditions (Figure 2a; Table 4). These results suggest that small-scale ecological conditions might be more important for shaping handling aggression phenotype than ecological conditions occurring at the landscape level. Our results thus suggest that, depending on the trait under study, personality phenotypes can be influenced by processes happening at different spatial scales (Quinn et al. 2009). More study sites with different degrees of ecological differences at varying spatial scales would be necessary to further explore this interesting phenomenon.

### Temporal variation in mean phenotype: selection or plasticity?

The yearly changes in mean phenotypes and the significant interaction between population and year for handling aggression, HR, and exploration speed (Table 3 and Supplementary Tables S8–S10) suggest 2 possibilities that are not mutually exclusive. The first possibility is that traits were plastic and their mean varied within a population according to local temporal variation in environmental conditions. Indeed, variation in environmental conditions may affect life-history characteristics, and thus personality traits, either directly through the plasticity of individuals or indirectly through maternal effects (Nicolaus et al. 2012; Montiglio et al. 2014). Second, yearly variation in environmental conditions may have created selection pressures (e.g., through differential mortality) that have led to short-term changes in the mean phenotypes within each population (Réale and Festa-Bianchet 2003; Dingemanse et al. 2004; Boon et al. 2007; Kontiainen et al. 2009; Quinn et al. 2009). In this case, we would expect that traits would not change from year to year within individuals, but that, instead, the populations in different years would be made up of different types of individuals. Exploring the relative importance of these 2 processes would require testing for within-individual changes in personality traits and personality-dependent demographic changes. These questions were not the goal of this study, but we can suggest a few explanations. In great tits, changes in population density and food abundance drive phenotypic changes in personality and selection pressures on behavioral traits (Dingemanse et al. 2004; Nicolaus et al. 2016). These factors may also shape personality in the closely related blue tit. Preliminary analyses in the 3 Corsican populations suggest that handling aggression was not affected by population density (Supplementary Figure S3) but depended on the amount of caterpillar frass (an indication of the caterpillar abundance; Supplementary Figure S4). Testing whether phenotypically plastic changes or personality-dependent survivals are responsible for yearly changes in the phenotypes measured would be an exciting follow-up but could only be done on a longer time scale. Whatever the reasons for these changes, these results indicate that among-population comparisons of pace-of-life characteristics should be made with caution if data are not collected over several years and under contrasting environmental conditions.

### Heart rate during manual restraint

Heart rate and breath rate reflect the activity of the sympathetic and parasympathetic nervous systems (Koolhaas et al. 1999). The sympathetic nervous system is suspected to be the dominant system in individuals that display fast exploration patterns, high handling aggression, and that exhibit a fast life-history strategy and invest more in current reproduction. The parasympathetic system is suspected to be the dominant system in slow exploring and docile individuals that exhibit a slower life-history strategy and invest more in future reproduction (proactive vs. reactive coping styles: Koolhaas et al. 1999; Réale et al. 2010; Ferrari et al. 2013). According to the pace-of-life syndrome hypothesis, the birds from the evergreen populations should exhibit a personality typical of a slow pace-of-life and thus a higher activity of the parasympathetic system and a slower heart rate during stressful events (Koolhaas et al. 1999; Ferrari et al. 2013). The tendency for a slower male heart rate in E-Muro than in D-Muro is in accordance with this prediction. However, contrary to our expectations, male heart rate was faster in E-Pirio then in E-Muro. This result contradicts the literature on pace-of-life and coping style (Koolhaas et al. 1999; Réale et al. 2010; Ferrari et al. 2013). However, a fast breath rate has been found to be associated with low activity in the novel-environment apparatus and with low handling aggression in other blue tit studies (Kluen et al. 2014; but see Fucikova et al. 2009). We found a positive relationship between breath rate and heart rate in our populations. Therefore, our results indicate that males in E-Pirio are less active in the novel-environment apparatus and have a potentially faster breath rate, which is in line with previous studies on blue tits personality (Kluen et al. 2014) even though it contradicts the general pace-of-life syndrome expectations (Réale et al. 2010). Further studies would be needed to clarify the association between the autonomous nervous system and both personality and life-history traits in avian species.

### Nest defense behavior

We found a significant repeatability for nest defense behavior (Table 2) revealing among-individual differences in nest defense in blue tits. We also found that birds in a pair showed positively correlated nest defense behavior. This correlation between partners could be caused by environmental factors shared by both parents, such as brood size (Montgomerie and Weatherhead 1988), or be the result of individuals matching their behavior to their partner’s (Schuett et al. 2010). Alternatively, this relationship could indicate behavioral assortative mating choice in these populations (Schuett et al. 2010; Class et al. 2014).

Nest defense behavior involves a trade-off between parental survival, energy reserve, and offspring protection (Trivers 1972; Montgomerie and Weatherhead 1988). Birds that have a lower future reproductive value and invest more in current reproduction should take more risks and invest more in offspring defense (Hakkarainen and Korpimäki 1994; Wolf et al. 2007; Møller and Nielsen 2014). Because they are faced with lower survival probability and larger clutches (Grosbois et al. 2006; Charmantier et al. 2016; Table 1), D-Muro birds were expected to approach the stuffed predator and the nest-box closer than birds from the evergreen populations. Contrary to this prediction, we did not find any difference among populations in nest defense behavior (Figure 2d). It is possible that, contrary to expectations (Wolf et al. 2007; Sih et al. 2015), risk taking during nest defense is not related to other measures of life-history characteristics in these blue tit populations. Alternatively, the correlation between risk taking during nest defense and other life-history traits could exist in our system but be detectable only at the within-population level if we compare individuals instead of populations (between-individuals correlation; Dingemanse and Dochtermann 2013).

### Sex-specific personality phenotypes

An increasing number of studies are showing sex differences in personality traits and behavioral syndromes (Schuett et al. 2010; Dammhahn 2012; Fresneau et al. 2014). For example, Fresneau et al. (2014) found different behavioral syndromes between males and females in a Finnish population of blue tits. We found sex-specific personality phenotypes in this study, with differences between sexes in mean phenotype for all traits and sex-specific difference between populations for heart rate during manual restraint. We also found that nest defense behavior was repeatable for females but not for males. In general, intersexual differences in personality phenotypes are not well understood, but likely arise because of intersexual differences in life-history strategies and selection pressures (Dingemanse et al. 2004; Dammhahn 2012; Class et al. 2014). A detailed investigation of sex-specific selection acting on these traits would help to explain the sexual dimorphism described in this study.

### Local adaptation in personality traits

Phenotypic differences between the 3 blue tit populations could be interpreted as divergent adaptations to habitat-specific ecological conditions, but from the present study, we cannot conclude whether these differences are due to behavioral plasticity or due to underlying genetic differences. However, several lines of evidence from recent studies on personality variation and past investigations in these populations suggest that differences in personality traits likely reflect genetic differences among populations and adaptations to local ecological conditions. First, personality in *Parus* is under selection (Dingemanse et al. 2004; Quinn et al. 2009; Nicolaus et al. 2016) and is heritable (Brommer and Kluen 2012; Class et al. 2014). Second, common-garden experiments have revealed genetic differences in life-history, morphological, and other behavioral traits among the 3 populations (Table 1; Blondel et al. 1999; Braillet et al. 2002; Charmantier et al. 2016). Third, genomic analyses using RAD sequencing have recently revealed a fine-scale genetic differentiation with a significant Fst of 1.8% between D-Muro and E-Muro (Porlier et al. 2012; Szulkin et al. 2016). Fourth, genetic drift is not likely to have driven such phenotypic differences, considering the very large population size (roughly estimated around 10000 in the Regino valley alone; Charmantier A, personal communication). Finally, preliminary results from a common-garden experiment suggest a genetic basis for the phenotypic differences between these populations in personality phenotypes (Dubuc-Messier G et al., in preparation).

### Conclusion and perspective

Our results reveal divergent personality phenotypes among 3 blue tit populations separated by spatial distances within the dispersal ability of the species and reveal strong temporal variation in mean personality phenotypes within populations. These populations inhabit areas with contrasting ecological conditions and display different life-history characteristics. This study thus emphasizes the role of environmental heterogeneity on behavioral diversity linked to life-history characteristics. An interesting next step would be to determine whether the phenotypic differences described across populations are mainly of genetic or environmental origin and whether these differences result from habitat-specific selection pressures and represent local adaptations. Different mechanisms could be responsible for fine-scale genetic differentiation for personality traits, among which matching habitat choice (Cote and Clobert 2007; Edelaar and Bolnick 2012), selective barriers against migrants, and positive assortative mating (Richardson et al. 2014) would be appealing possibilities for future research. Furthermore, these Corsican blue tit populations are located at the extreme south of the blue tit distribution and, based on their small clutch size, they are located on the slower end of the pace-of-life continuum. An interesting and broader approach to study the pace-of-life syndrome hypothesis would thus be to compare populations at a much larger scale by including populations located further north within the species’ range.

## SUPPLEMENTARY MATERIAL

Supplementary material can be found at http://www.beheco.oxfordjournals.org/

## FUNDING

This work was supported by the Agence Nationale de la Recherche (BioAdapt grant ANR-12-ADAP-0006-02-PEPS to A.C.), the European Research Council (Starting grant ERC-2013-StG-337365-SHE to A.C.), the Observatoire des Science de l’Univers—Observatoire de Recherche Méditerranéen de l’Environnement to A.C., and the Natural Sciences and Engineering Research Council of Canada (NSERC; Discovery Grant) to D.R. G.D.M. received a PhD fellowship from the Fonds de Recherche Québec Nature et Technologies and of Natural Science and Engineering Research Council of Canada.

## Supplementary Material

Supplementary_Material
