# Supplementary material for: Environmental heterogeneity and population differences in blue tits personality traits
Source: Behav Ecol. 2016 Dec 20;28(2):448–59. doi: 10.1093/beheco/arw148 (PMC5873839; doi:10.1093/beheco/arw148)
Supplement: Supplementary_Material [file arw148_suppl_supplementary_material.doc]

**Supplementary materials**

Number of observers

**Table S1. Total number of handling aggression observations per observer, year and population for blue tits in Corsica (France); there are an additional 13 observations from an unknown observer.**

| Observers | D-Muro | E-Muro | E-Pirio | Total |  | Observers | D-Muro | E-Muro | E-Pirio | Total |
| --- | --- | --- | --- | --- | --- | --- | --- | --- | --- | --- |
| **AC Total** | 68 | 25 | 15 | 108 |  | **MS Total** | 0 | 0 | 12 | 12 |
| 2011 | 25 | 19 | 4 | 48 |  | 2011 | 0 | 0 | 0 | 0 |
| 2012 | 35 | 3 | 0 | 38 |  | 2012 | 0 | 0 | 0 | 0 |
| 2013 | 1 | 1 | 4 | 6 |  | 2013 | 0 | 0 | 0 | 0 |
| 2014 | 7 | 2 | 7 | 16 |  | 2014 | 0 | 0 | 12 | 12 |
|  |  |  |  |  |  |  |  |  |  |  |
| **CD Total** | 0 | 0 | 82 | 82 |  | **OJ Total** | 0 | 0 | 8 | 8 |
| 2011 | 0 | 0 | 1 | 18 |  | 2011 | 0 | 0 | 8 | 8 |
| 2012 | 0 | 0 | 23 | 23 |  | 2012 | 0 | 0 | 0 | 0 |
| 2013 | 0 | 0 | 23 | 23 |  | 2013 | 0 | 0 | 0 | 0 |
| 2014 | 0 | 0 | 18 | 18 |  | 2014 | 0 | 0 | 0 | 0 |
|  |  |  |  |  |  |  |  |  |  |  |
| **CF Total** | 138 | 92 | 99 | 329 |  | **PAM Total** | 28 | 38 | 30 | 96 |
| 2011 | 0 | 0 | 0 | 0 |  | 2011 | 0 | 0 | 0 | 0 |
| 2012 | 62 | 39 | 40 | 141 |  | 2012 | 12 | 15 | 0 | 27 |
| 2013 | 10 | 11 | 30 | 51 |  | 2013 | 16 | 21 | 17 | 54 |
| 2014 | 66 | 42 | 29 | 137 |  | 2014 | 0 | 2 | 13 | 15 |
|  |  |  |  |  |  |  |  |  |  |  |
| **CGE Total** | 23 | 18 | 18 | 59 |  | **PP Total** | 188 | 123 | 155 | 466 |
| 2011 | 0 | 0 | 0 | 0 |  | 2011 | 56 | 54 | 100 | 210 |
| 2012 | 0 | 0 | 0 | 0 |  | 2012 | 46 | 27 | 52 | 125 |
| 2013 | 0 | 0 | 0 | 0 |  | 2013 | 31 | 12 | 3 | 46 |
| 2014 | 23 | 18 | 18 | 59 |  | 2014 | 55 | 30 | 0 | 85 |
|  |  |  |  |  |  |  |  |  |  |  |
| **DR Total** | 12 | 5 | 0 | 17 |  | **VJ Total** | 37 | 9 | 23 | 69 |
| 2011 | 0 | 0 | 0 | 0 |  | 2011 | 0 | 0 | 0 | 0 |
| 2012 | 0 | 0 | 0 | 0 |  | 2012 | 0 | 0 | 0 | 0 |
| 2013 | 0 | 1 | 0 | 1 |  | 2013 | 37 | 9 | 23 | 69 |
| 2014 | 12 | 4 | 0 | 16 |  | 2014 | 0 | 0 | 0 | 0 |
|  |  |  |  |  |  |  |  |  |  |  |
| **EM Total** | 19 | 28 | 32 | 79 |  | **SAC Total** | 0 | 0 | 25 | 25 |
| 2011 | 19 | 28 | 32 | 79 |  | 2011 | 0 | 0 | 25 | 25 |
| 2012 | 0 | 0 | 0 | 0 |  | 2012 | 0 | 0 | 0 | 0 |
| 2013 | 0 | 0 | 0 | 0 |  | 2013 | 0 | 0 | 0 | 0 |
| 2014 | 0 | 0 | 0 | 0 |  | 2014 | 0 | 0 | 0 | 0 |
|  |  |  |  |  |  |  |  |  |  |  |
| **GDM Total** | 48 | 53 | 62 | 163 |  |  |  |  |  |  |
| 2011 | 0 | 0 | 0 | 0 |  |  |  |  |  |  |
| 2012 | 24 | 8 | 25 | 57 |  |  |  |  |  |  |
| 2013 | 18 | 27 | 19 | 64 |  |  |  |  |  |  |
| 2014 | 6 | 18 | 18 | 42 |  |  |  |  |  |  |
|  |  |  |  |  |  |  |  |  |  |  |
| **JB Total** | 43 | 21 | 0 | 64 |  |  |  |  |  |  |
| 2011 | 0 | 0 | 0 | 0 |  |  |  |  |  |  |
| 2012 | 18 | 9 | 0 | 27 |  |  |  |  |  |  |
| 2013 | 25 | 12 | 0 | 37 |  |  |  |  |  |  |
| 2014 | 0 | 0 | 0 | 0 |  |  |  |  |  |  |
|  |  |  |  |  |  |  |  |  |  |  |
| **KD Total** | 0 | 0 | 22 | 22 |  |  |  |  |  |  |
| 2011 | 0 | 0 | 0 | 0 |  |  |  |  |  |  |
| 2012 | 0 | 0 | 0 | 0 |  |  |  |  |  |  |
| 2013 | 0 | 0 | 0 | 0 |  |  |  |  |  |  |
| 2014 | 0 | 0 | 22 | 22 |  |  |  |  |  |  |
|  |  |  |  |  |  |  |  |  |  |  |
| **MOB Total** | 51 | 17 | 31 | 99 |  |  |  |  |  |  |
| 2011 | 0 | 0 | 0 | 0 |  |  |  |  |  |  |
| 2012 | 0 | 0 | 0 | 0 |  |  |  |  |  |  |
| 2013 | 30 | 2 | 20 | 52 |  |  |  |  |  |  |
| 2014 | 21 | 15 | 11 | 47 |  |  |  |  |  |  |
|  |  |  |  |  |  |  |  |  |  |  |
| **MP Total** | 59 | 21 | 0 | 80 |  |  |  |  |  |  |
| 2011 | 59 | 21 | 0 | 80 |  |  |  |  |  |  |
| 2012 | 0 | 0 | 0 | 0 |  |  |  |  |  |  |
| 2013 | 0 | 0 | 0 | 0 |  |  |  |  |  |  |
| 2014 | 0 | 0 | 0 | 0 |  |  |  |  |  |  |

Table S2. Total number of heart rate observations per observer, year and population for blue tits in Corsica (France); there are an additional 17 observations from an unknown observer.

| Observers | D-Muro | E-Muro | E-Pirio | Total |
| --- | --- | --- | --- | --- |
| **AC Total** | 51 | 46 | 4 | 101 |
| 2011 | 22 | 26 | 4 | 52 |
| 2012 | 29 | 15 | 0 | 44 |
| 2013 | 0 | 5 | 0 | 5 |
| 2014 | 0 | 0 | 0 | 0 |
| 2015 | 0 | 0 | 0 | 0 |
|  |  |  |  |  |
| **DR Total** | 33 | 10 | 0 | 43 |
| 2011 | 2 | 3 | 0 | 5 |
| 2012 | 0 | 0 | 0 | 0 |
| 2013 | 28 | 4 | 0 | 32 |
| 2014 | 3 | 3 | 0 | 6 |
| 2015 | 0 | 0 | 0 | 0 |
|  |  |  |  |  |
| **GDM Total** | 43 | 46 | 82 | 171 |
| 2011 | 0 | 0 | 0 | 0 |
| 2012 | 10 | 7 | 35 | 52 |
| 2013 | 6 | 4 | 11 | 21 |
| 2014 | 6 | 7 | 4 | 17 |
| 2015 | 21 | 28 | 32 | 81 |
|  |  |  |  |  |
| **PP Total** | 6 | 9 | 0 | 15 |
| 2011 | 6 | 9 | 0 | 15 |
| 2012 | 0 | 0 | 0 | 0 |
| 2013 | 0 | 0 | 0 | 0 |
| 2014 | 0 | 0 | 0 | 0 |
| 2015 | 0 | 0 | 0 | 0 |
|  |  |  |  |  |
| **SAC Total** | 0 | 0 | 19 | 19 |
| 2011 | 0 | 0 | 19 | 19 |
| 2012 | 0 | 0 | 0 | 0 |
| 2013 | 0 | 0 | 0 | 0 |
| 2014 | 0 | 0 | 0 | 0 |
| 2015 | 0 | 0 | 0 | 0 |

Table S3. Total number of nest defense observations per observer, year and population for blue tits in Corsica (France); all observers were identified.

| Observers | D-Muro | E-Muro | E-Pirio | Total |
| --- | --- | --- | --- | --- |
| **CF Total** | 0 | 0 | 4 | 4 |
| 2012 | 0 | 0 | 0 | 0 |
| 2013 | 0 | 0 | 0 | 0 |
| 2014 | 0 | 0 | 0 | 0 |
| 2015 | 0 | 0 | 4 | 4 |
| **CGE Total** | 29 | 26 | 13 | 68 |
| 2012 | 0 | 0 | 0 | 0 |
| 2013 | 0 | 0 | 0 | 0 |
| 2014 | 29 | 26 | 13 | 68 |
| 2015 | 0 | 0 | 0 | 0 |
| **GDM Total** | 31 | 24 | 58 | 113 |
| 2012 | 0 | 0 | 15 | 15 |
| 2013 | 0 | 0 | 17 | 17 |
| 2014 | 27 | 20 | 18 | 65 |
| 2015 | 4 | 4 | 8 | 16 |
| **MOB Total** | 0 | 0 | 33 | 33 |
| 2012 | 0 | 0 | 0 | 0 |
| 2013 | 0 | 0 | 0 | 0 |
| 2014 | 0 | 0 | 33 | 33 |
| 2015 | 0 | 0 | 0 | 0 |
| **RMG Total** | 6 | 5 | 5 | 16 |
| 2012 | 0 | 0 | 0 | 0 |
| 2013 | 0 | 0 | 0 | 0 |
| 2014 | 0 | 0 | 0 | 0 |
| 2015 | 6 | 5 | 5 | 16 |
| **SCB Total** | 7 | 8 | 17 | 32 |
| 2012 | 0 | 0 | 0 | 0 |
| 2013 | 0 | 0 | 0 | 0 |
| 2014 | 0 | 0 | 0 | 0 |
| 2015 | 7 | 8 | 17 | 32 |
| **VJ Total** | 0 | 0 | 15 | 15 |
| 2012 | 0 | 0 | 0 | 0 |
| 2013 | 0 | 0 | 15 | 15 |
| 2014 | 0 | 0 | 0 | 0 |
| 2015 | 0 | 0 | 0 | 0 |

Handling aggression scoring protocol

Table S4: Blue tit handling aggression scale

| Score | Wings spread | Tail feathers spread | Bird strikes fingers |
| --- | --- | --- | --- |
| 0 | No | No | No |
| 1 | No | No | Yes, but only if provoked |
| 2 | No | Yes | Yes, spontaneously |
| 3 | Yes | Yes | Yes, spontaneously |

When the bird displayed one reaction specific to one score and another reaction specific to another score, it received an average score between the two. For example, a bird that struck without any provocation (score 2) but did not have its wings and tail feathers spread (score 1) would be scored as 1.5.

Repeatability estimates for handling aggression scores for the two periods of capture

Table S5. Adjusted repeatability estimates (*r_ID_*) for handling aggression scores for the two periods of capture (among-years for the pre-breeding and breeding period and within-year among periods for every year (2011 to 2014)) for three blue tit populations in Corsica (France).

| Period / Year |  | *r_ID_* | L-ratio | p-value | N_Obs_; N_ID_ |
| --- | --- | --- | --- | --- | --- |
| *Among years, within period* |  |  |  |  |  |
|  | Pre-breeding | 0.21 | 7.877 | < 0.010 | 615; 491 |
|  | Breeding | 0.32 | 41.290 | < 0.001 | 1083; 754 |
| *Within year, among periods* |  |  |  |  |  |
|  | 2011 | 0.21 | 4.036 | < 0.05 | 412; 320 |
|  | 2012 | 0.33 | 14.520 | < 0.001 | 431; 331 |
|  | 2013 | 0.35 | 13.930 | < 0.001 | 389; 302 |
|  | 2014 | 0.14 | 2.010 | 0.16 | 466; 353 |

L-ratio and p-values represent the results from the comparison of a full model and a model without individual identity in random effect. N_Obs_ refers to the number of observations, N_ID_ refers to the number of different individuals considered in the models. Data from the three populations and for both sexes were included. All significant fixed effects were included in the models as detailed in Table S8 to S11.

Repeatability estimates for each population and sex

Table S6. Variance components (with 95% confidence intervals) and adjusted repeatability estimates (*r*_ID_) for three personality traits and one physiological trait for each blue tit population in Corsica (France).

| Trait | Population | V_ID_ (CI) | V_OBS_ (CI) | V_R_ (CI) | *r*_ID_  (N_Obs_; *N_ID_*) | L-ratio | p-value |
| --- | --- | --- | --- | --- | --- | --- | --- |
| *Handling aggression* | D-Muro | 0.23  (0.15; 0.32) | 0.01  (0.000; 0.04) | 0.53  (0.45; 0.60) | 0.25  (703; 365) | 49.398 | < 0.001 |
|  | E-Muro | 0.18  (0.06; 0.31) | 0.03  (0.000; 0.11) | 0.68  (0.56; 0.82) | 0.20  (447; 238) | 10.880 | < 0.001 |
|  | E-Pirio | 0.25  (0.14; 0.37) | 0.04  (0.006; 0.11) | 0.65  (0.55; 0.76) | 0.26  (549; 295) | 28.093 | < 0.001 |
| *HR (beats/min.)* | D-Muro | 5884.50  (2912.90; 8033.22) | 524.40  (0.000; 2053.92) | 2274.30  (1301.97; 4357.15) | 0.68  (143; 114) | 15.18 | < 0.001 |
|  | E-Muro | 10620.00  (5183.04; 15434.47) | 1.46x10^-10^  (0.000; 851.09) | 3642.00  (1735.36; 6899.64) | 0.75  (116; 92) | 8.244 | < 0.005 |
|  | E-Pirio | 6389.0  (4297.60; 8508.72) | 0.00  (0.000; 381.88) | 726.60  (319.28; 1322.37) | 0.90  (107; 89) | 31.352 | < 0.001 |
| *Average exploration speed (cm/s)* | D-Muro | 29.45  (13.78; 45.16) | N.A. | 22.12  (13.92; 37.24) | 0.57  (175; 143) | 12.022 | < 0.001 |
|  | E-Muro | 18.42  (4.93; 32.09) | N.A. | 15.63  (8.42; 30.68) | 0.54  (100; 86) | 4.662 | < 0.050 |
|  | E-Pirio | 12.82  (0.00; 37.24) | N.A. | 34.93  (17.43; 61.05) | 0.27  (105; 89) | 0.844 | 0.33 |

L-ratio and p-values represent the results from the comparison of a full model and a model without individual identity as random effect. N_Obs_ refers to the number of observations and N_ID_ to the number of different individuals considered in the models. All significant fixed effects for each trait were included in the models as detailed in Table S8 to S11.

Table S7. Sex-specific variance components (with 95% confidence intervals) and adjusted repeatability estimates (*r*_ID_) for three personality traits in blue tits from Corsica (France).

| Trait | Sex | V_ID_ (CI) | V_OBS_ (CI) | V_R_ (CI) | *r*_ID_  (N_Obs_;*N_ID_*) | L-ratio | p-value |
| --- | --- | --- | --- | --- | --- | --- | --- |
| *Handling aggression* | F | 0.24  (0.16; 0.32) | 0.02  (0.00; 0.07) | 0.62  (0.54; 0.69) | 0.27  (886; 457) | 51.005 | < 0.001 |
|  | M | 0.21  (0.12; 0.30) | 0.04  (0.01; 0.11) | 0.59  (0.51; 0.68) | 0.25  (813; 449) | 30.117 | < 0.001 |
| *HR (beats/min.)* | F | 8963.90  (6201.90;1193.89) | 788.80  (0.00; 2190.87) | 2157.30  (1014.46;3296.18) | 0.75  (199; 157) | 25.498 | < 0.001 |
|  | M | 8108.90  (5726.19;9949.06) | 3643.30  (0.00; 10035.68) | 479.90  (185.92;1069.49) | 0.66  (167; 140) | 33.127 | < 0.001 |
| *Average exploration speed (cm/s)* | F | 21.52  (7.34; 34.44) | N.A. | 26.78  (15.39; 38.27) | 0.45  (208; 173) | 7.852 | < 0.010 |
|  | M | 18.66  (2.46; 33.64) | N.A. | 30.52  (15.96; 44.70) | 0.38  (171; 147) | 4.346 | < 0.05 |
| *Nest defense (m)* | F | 0.25  (0.09; 0.39) | 0.16  (0.02; 0.55) | 0.18  (0.07; 0.32) | 0.42  (141; 125) | 8.4257 | < 0.005 |
|  | M | 0.042  (0.00; 0.14) | 0.10  (0.01; 0.33) | 0.27  (0.16; 0.37) | 0.25  (144; 115) | 1.985 | 0.55 |

F : female, M: males; L-ratio and p-values represent the results from the comparison of a full model and a model without individual identity in random effect. N_Obs_ refers to the number of observations and N_ID_  to the number of different individuals considered in the models. Data from all years and the three populations were included. All the significant fixed effects for each trait were included in models as detailed in Table S8 to S11.

Finals models describing each personality trait (estimates and confidence intervals)

Table S8. Final model describing the handling aggression scores measured in three blue tit populations in Corsica (France) between 2011 and 2014; estimates and confidence intervals (CI 95%) are presented for each term.

| Terms | Estimates | Lower  Confidence intervals | Upper  Confidence intervals |
| --- | --- | --- | --- |
| Intercept | 2.29 | 1.99 | 2.59 |
| Pop.: E-Muro | -0.77 | -0.99 | -0.54 |
| Pop.: E-Pirio | -0.88 | -1.11 | -0.66 |
| Sex: Females | -0.34 | -0.44 | -0.24 |
| Time of day | 0.01 | -0.01 | 0.02 |
| Capture period: Breeding | 0.06 | -0.02 | 0.19 |
| Capture rank | 0.02 | -0.01 | 0.05 |
| Year: 2012 | -0.79 | -0.99 | -0.59 |
| Year: 2013 | -0.90 | -1.12 | -0.68 |
| Year: 2014 | -0.80 | -1.01 | -0.58 |
| Pop.: E-Muro* Year: 2012 | 0.70 | 0.43 | 1.04 |
| Pop.: E-Muro* Year: 2013 | 0.68 | 0.37 | 1.00 |
| Pop.: E-Muro* Year: 2014 | 0.69 | 0.10 | 0.39 |
| Pop.: E-Pirio* Year: 2012 | 0.95 | 0.69 | 1.26 |
| Pop.: E-Pirio* Year: 2013 | 1.05 | 0.77 | 1.36 |
| Pop.: E-Pirio* Year: 2014 | 0.88 | 0.58 | 1.18 |

References: Population: D-Muro; Sex: Males; Capture period: Pre-breeding; Year: 2011.

Table S9. Final model describing mean heart rate during manual restraint (HR; in beats/min.) measured in three blue tit populations in Corsica (France) between 2011 and 2015; estimates and confidence intervals (CI 95%) are presented for each term.

| Terms | Estimates | Lower  Confidence intervals | Upper  Confidence intervals |
| --- | --- | --- | --- |
| Intercept | 936.74 | 714.10 | 1157.72 |
| Pop.: E-Muro | 26.95 | -18.58 | 72.41 |
| Pop.: E-Pirio | 26.05 | -28.33 | 80.48 |
| Year: 2012 | 4.29 | -35.72 | 43.90 |
| Year: 2013 | 7.42 | -40.82 | 55.33 |
| Year: 2014 | 48.89 | -14.67 | 127.90 |
| Year: 2015 | 51.03 | -3.99 | 105.63 |
| Sex: Females | 5.76 | -16.92 | 28.36 |
| Time of day | 0.29 | -3.54 | 4.16 |
| Body mass | 1.63 | -21.53 | 24.86 |
| Time between capture and recording | -109.43 | -193.39 | -25.55 |
| Pop.: E-Muro* Year: 2012 | -41.36 | -95.24 | 13.51 |
| Pop.: E-Muro* Year: 2013 | -9.27 | -78.43 | 60.80 |
| Pop.: E-Muro* Year: 2014 | -53.19 | -135.98 | 31.05 |
| Pop.: E-Muro* Year: 2015 | -83.01 | -151.39 | -14.26 |
| Pop.: E-Pirio* Year: 2012 | 39.60 | -27.67 | 106.10 |
| Pop.: E-Pirio* Year: 2013 | 5.09 | -78.96 | 89.26 |
| Pop.: E-Pirio* Year: 2014 | -109.06 | -216.86 | -0.45 |
| Pop.: E-Pirio* Year: 2015 | -57.68 | -132.34 | 16.90 |

Reference: Population: D-Muro ; Sex: Males; Year: 2011.

Table S10. Final model describing the average exploration speed (cm/s) for blue tits in Corsica (France) between 2011 and 2014; estimates and confidence intervals (CI 95%) are presented for each term.

| Terms | Estimates | Lower  Confidence intervals | Upper  Confidence intervals |
| --- | --- | --- | --- |
| Intercept | 17.62 | 13.53 | 21.69 |
| Pop.: E-Muro | -2.90 | -6.13 | 0.33 |
| Pop.: E-Pirio | -5.58 | -9.10 | -2.04 |
| Sex: Females | -2.02 | -3.49 | -0.55 |
| Contention method: bag | 1.24 | -1.11 | 3.60 |
| Contention method: cage | -2.21 | -3.92 | -0.50 |
| Time of day | -0.05 | -0.31 | 0.21 |
| Year: 2012 | 0.69 | -2.25 | 3.63 |
| Year: 2013 | 0.40 | -2.73 | 3.53 |
| Year: 2014 | -8.64 | -11.73 | -5.57 |
| Pop.: E-Muro*Year: 2012 | 2.75 | -1.56 | 7.06 |
| Pop.: E-Muro*Year: 2013 | -0.17 | -5.16 | 4.82 |
| Pop.: E-Muro*Year: 2014 | 2.84 | -1.69 | 7.38 |
| Pop.: E-Pirio*Year: 2012 | -0.06 | -4.65 | 4.48 |
| Pop.: E-Pirio*Year: 2013 | 2.66 | -2.00 | 7.29 |
| Pop.: E-Pirio*Year: 2014 | 6.81 | 2.03 | 11.60 |

Reference for every term: population: D-Muro; Sex: Males; Contention methods: no contention; Year: 2011.

Table S11. Final model describing the minimal approach distance during nest defense trials for blue tits in Corsica (France) between 2011 and 2015; estimates and confidence intervals (CI 95%) are presented for each term.

| Terms | Estimates | Lower  Confidence intervals | Upper  Confidence intervals |
| --- | --- | --- | --- |
| Intercept | 2.28 | 1.50 | 3.05 |
| Year: 2013 | -0.75 | -1.14 | -0.33 |
| Year: 2014 | -0.50 | -0.82 | -0.17 |
| Year: 2015 | -0.81 | -1.21 | -0.39 |
| Sex: Females | 0.20 | 0.05 | 0.35 |
| Time of day | 0.02 | -0.05 | 0.09 |
| Identity of the decoy: no2 | 0.20 | -0.40 | 0.80 |
| Dist. between decoy  and nest-box | 0.001 | -0.0002 | 0.002 |
| Dist. to closest branch | -0.001 | -0.002 | 0.0003 |
| Observer dist.  from nest-box | 0.05 | -0.03 | 0.13 |

Reference: Year: 2011, Sex: Males, Identity of the decoy: no1

Table S12. Difference between birds from the Fango and Regino valley in handling aggression score and average exploration speed for blue tits in Corsica (France) between 2011 and 2014; we used the same fixed effect structure as for the models with population as a fixed effect (using valley instead of population; but without the interaction terms between valley and year, see Table S8 and S10).

| Trait | Estimates  Fango valley | 95% CI | L-ratio | p-value |
| --- | --- | --- | --- | --- |
| Handling aggression | -0.054 | -0.173; 0.066 | 0.782 | 0.37 |
| Average exploration speed | -2.803 | -4.493; -1.114 | 10.479 | < 0.001 |

Reference: Regino valley

The novel-environment apparatus

a)


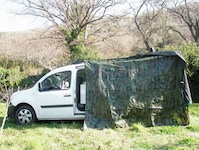

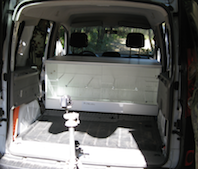


b)


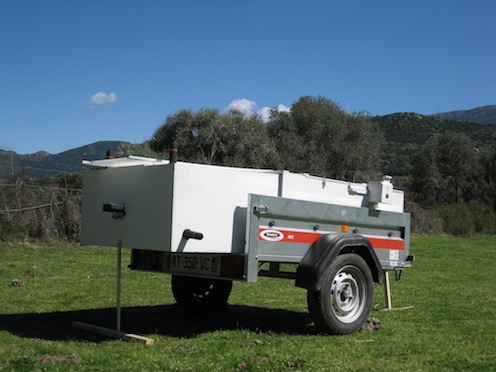

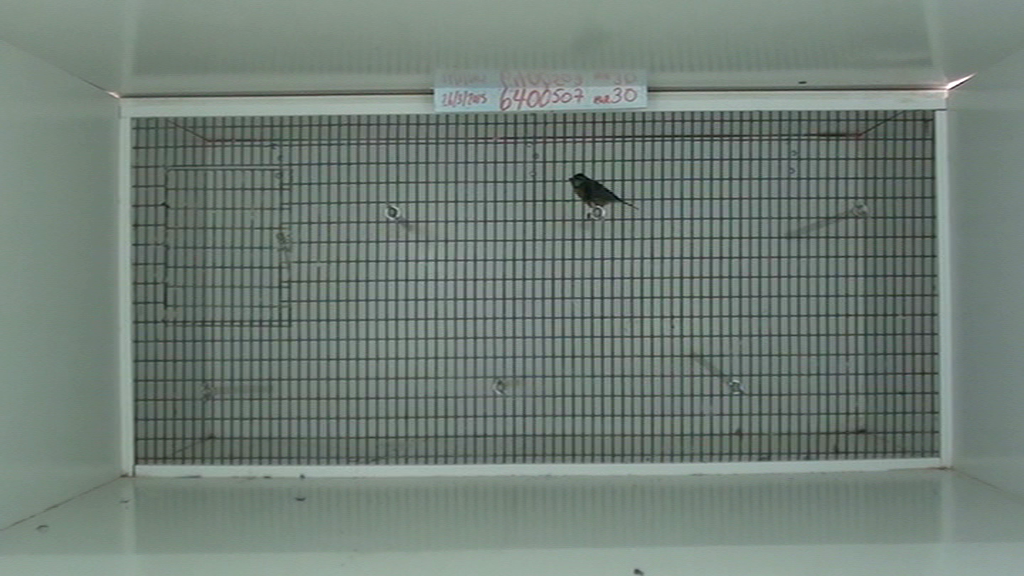


Figure S1: Novel-environment apparatus used in a) 2011 to 2013 and b) 2014, view of the entire apparatus and of the cages.

Average personality phenotype for each year and population

a)


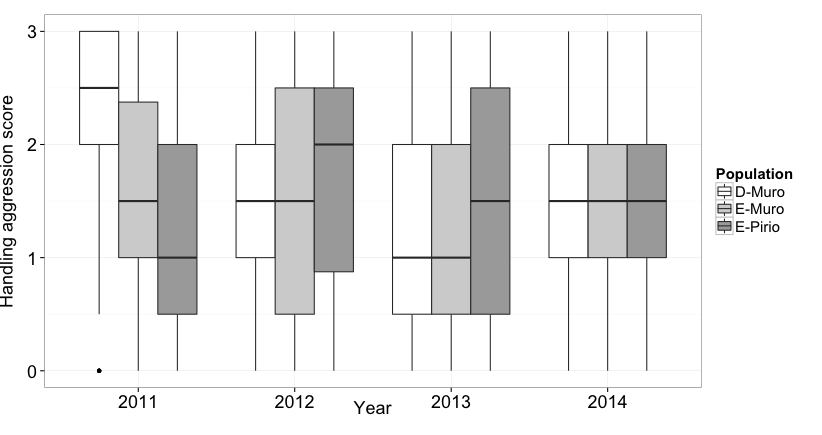


b)


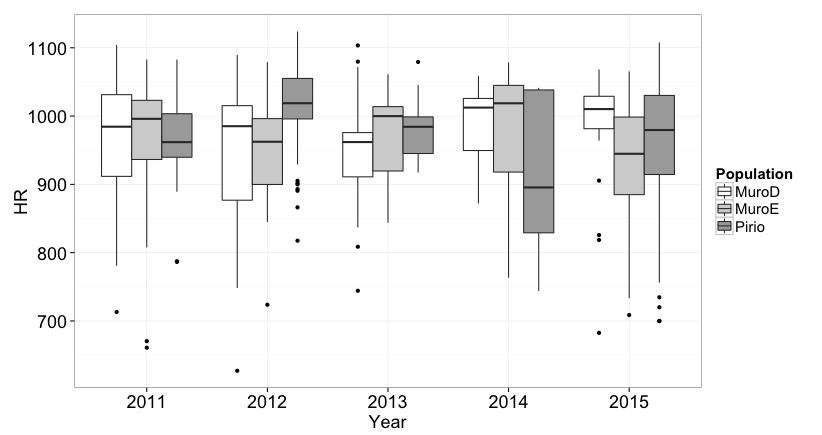


c)


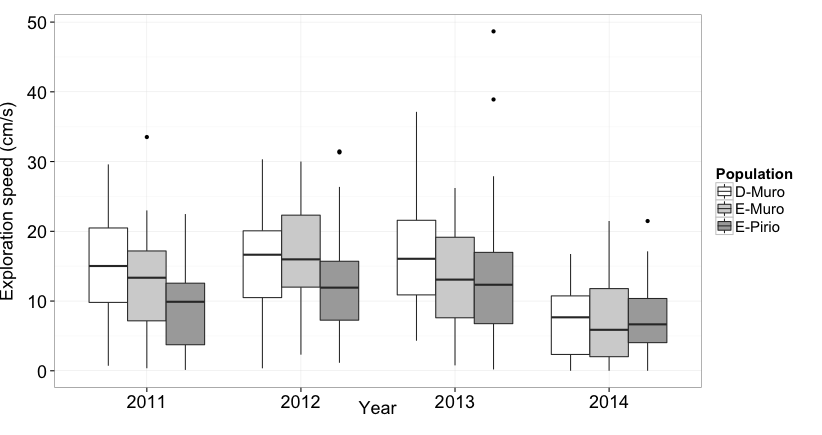


Figure S2. Average a) handling aggression score, b) heart rate during manual restraint (beats/min.), c) average exploration speed (cm/s) for each year and population for three Corsican blue tits populations (France).

Variation in mean handling aggression score in function of density and food abundance in each population

Figure S3. Variation across populations in mean handling aggression scores and proportion of occupied nest-boxes (as an indication of density). Blue and circle: D-Muro, black and squares: E-Muro and red and triangle: E-Pirio. A simple model with mean handling aggression for each population as response variable and as fixed effect the % of occupied nest-box and year, population and the interaction between population and % occupied nest-boxes reveals no effect of nest-box occupancy on mean handling aggression score and no interaction between population and % of occupied nest-boxes.

Figure S4. Variation across populations in mean handling aggression scores and maximal mg of caterpillar frass per m^2^ per day (see Zandt et al. 1990 for details about the caterpillar frass sampling procedure). Blue and circle: D-Muro, black and square: E-Muro and red and triangle: E-Pirio. In D-Muro, caterpillar abundance was positively related with the average population handling aggression (correlation estimate: 0.83). We found the opposite trend in E-Muro and E-Pirio: mean handling aggression scores increase with decreasing frass weight (cor : E-Muro :-0.79, E-Pirio :-0.96). A simple model with mean handling aggression for each population as response variable and as fixed effect the maximum of caterpillar frass, year, population and the interaction between population and max. caterpillar frass reveals a marginally significant interaction between population and caterpillar frass: the relationship between max. frass and mean handling aggression was opposite in E-Pirio and D-Muro (p-value = 0.060). The amount of caterpillar frass and the mean handling aggression scores were both particularly high in 2011 in D-Muro, which might explain the difference in handling aggression phenotypes between E-Pirio and D-Muro for this year in specificaly.

References

Zandt H, Strijkstra A, Blondel J, van Balen H. 1990. Food in two Mediterranean Blue Tit populations: Do differences in caterpillar availability explain differences in timing of the breeding season? In Blondel J, Gosler A, Lebreton JD, McCleery R, eds. Population Biology of Passerine Birds: An Integrated Approach. Berlin: Springer-Verlag. Pages 145–155.
